# The effect of exercise on blood concentrations of angiogenesis markers in older adults: a systematic review and meta-analysis

**DOI:** 10.21203/rs.3.rs-2468576/v1

**Published:** 2023-01-16

**Authors:** Bing Xin Song, Laiba Azhar, Grace Ka Yi Koo, Susan Marzolini, Damien Gallagher, Walter Swardfager, Clara Chen, Joycelyn Ba, Nathan Herrmann, Krista Lanctôt

**Affiliations:** University of Toronto; Sunnybrook Research Institute; Sunnybrook Research Institute; University Health Network; Sunnybrook Health Sciences Centre; Sunnybrook Research Institute; University of Toronto; University of Toronto; Sunnybrook Research Institute; Sunnybrook Research Institute

**Keywords:** Vascular endothelial growth factor, E-selectin, exercise, blood, angiogenesis

## Abstract

**Background:**

Physical exercise has positive impacts on health and can improve angiogenesis, which is impaired during aging, but the underlying mechanisms of benefit are unclear. This meta-analysis and systematic review investigated the effects of exercise on several peripheral angiogenesis markers in older adults to better understand the relationship between exercise and angiogenesis.

**Methods:**

MEDLINE, Embase, and Cochrane CENTRAL were searched for original, peer-reviewed reports of peripheral concentrations of angiogenesis markers before and after exercise interventions in older adults (> 50 years). The risk of bias was assessed with standardized criteria. Standardized mean differences (SMD) with 95% confidence intervals (CIs) were calculated from random-effects models. Publication bias was assessed with Egger’s test, funnel plots, and trim-and-fill. *A priori* subgroup analyses and meta-regressions were performed to investigate heterogeneity where possible.

**Results:**

Of the 44 articles included in the review, 38 were included in meta-analyses for five proteins. Vascular endothelial growth factor (VEGF) was found to be higher after exercise (SMD[95%CI] = 0.18[0.03, 0.34], p = 0.02), and e-selectin (CD62E) was found to be lower after exercise (SMD[95%CI]= −0.72[−1.42, −0.03], p = 0.04). Endostatin (SMD[95%CI] = 0.28[−0.56, 1.11], p = 0.5), fibroblast growth factor 2 (SMD[95%CI] = 0.03[−0.18, 0.23], p = 0.8), and matrix metallopeptidase-9 (SMD[95%CI] = −0.26[−0.97, 0.45], p = 0.5) levels did not change after exercise.

**Conclusions:**

Of the five angiogenesis blood markers evaluated in this meta-analysis, only VEGF and CD62E changed with exercise. Although more studies are needed, changes in angiogenesis markers may explain the beneficial effects of exercise on angiogenesis and health in older adults.

## Background/introduction

Angiogenesis is the process of growth and formation of new blood vessels from pre-existing vessels, helping to transport nutrients and proteins throughout the body. It requires the complex and coordinated activity of different growth factors, cell adhesion molecules, and metabolites in endothelial cells [1, 2]. There are several known angiogenesis markers, such as vascular endothelial growth factor (VEGF) and fibroblast growth factor 2 (FGF2) that promote angiogenesis, and anti-angiogenic factors, such as endostatin which competitively inhibits VEGF and increases endothelial cell apoptosis [3, 4]. For e-selectin (CD62E), although its role in angiogenesis has not been fully elucidated, it has been suggested to regulate the anti-angiogenic activity of endostatin [5] and angiostatin, another potent angiogenesis inhibitor[6]. Interestingly, metallopeptidase-9 (MMP9) is considered a pro-angiogenic factor that plays a crucial role in the modeling of the extracellular matrix, but it is also involved in the generation of the anti-angiogenic factor, angiostatin [7]. Importantly, several of these angiogenesis markers have been shown to change with age; VEGF and FGF2 can decrease with age, while matrix metalloproteinases can increase [8]. Moreover, the age-related impairment in angiogenesis can be associated with increased risk of cardiovascular diseases [9]. Considering the impairment of angiogenesis during aging [8, 10, 11], exploring interventions that can improve angiogenesis can be particularly important in older adults.

In older adults, exercise has a positive impact on overall health and wellness [12], with particular benefits on mental health and cardiovascular fitness [13]. Long-term physical activity of at least moderate intensity has been shown to improve cognitive outcomes in older adults over 50 years old [14]. However, the mechanisms via which exercise confers health benefits in older adults have not yet been fully elucidated. Physical exercise has been shown to improve angiogenesis in animals and humans [11, 15-18]. Although angiogenesis marker levels may return to baseline levels within a short interval (about 20 minutes) following acute aerobic and resistance exercise [19], long-term exercise have been associated with sustained alteration in angiogenesis marker levels and thus, may have more pronounced benefits for older adults [20]. Several reviews have suggested that exercise can change angiogenesis marker levels in healthy adults and even potentially older adults [11, 15-18], but the findings from exercise studies with angiogenesis markers are inconsistent and no meta-analysis has yet evaluated the impact of exercise on multiple angiogenesis blood markers in apparently healthy older adults. The aim of this systematic review and meta-analysis was to determine the effect of exercise on various angiogenesis blood markers in generally healthy older adults. This will allow for a better understanding of the angiogenesis processes following physical exercise in older adults.

## Methods

### Data Sources and Search Strategy

This systematic review was conducted according to our pre-defined protocol registered on the International Prospective Register of Systematic Reviews (CRD42022334061), following the Preferred Reporting Items for Systematic Reviews and Meta-Analysis guidelines [21]. English-language literature was searched on March 10, 2022, in Cochrane Central, Embase, and MEDLINE for original peer-reviewed articles. A sample search strategy is included in Additional file 1. Reference lists of included articles were further examined for additional eligible studies. Search strategies for all databases included key and MeSH terms with exercise and key angiogenesis proteins of interest.

### Study Selection

Inclusion criteria were as follows: 1) healthy older adults (including overweight and obese older adults); 2) ≥ 50 years of age (mean), or if mean age was not reported, population description implied that the group consisted of participants mostly ≥ 50 years of age; 3) measured serum, plasma or blood concentrations of selected angiogenesis proteins (Additional file 1) before and after an exercise intervention; and 4) the exercise intervention was at least moderate intensity (e.g. ≥40 to 59% heart rate reserve (HRR) or oxygen consumption reserve ($\dot{V}O_{2}R$), 46 to 63% of maximal oxygen consumption ($\dot{V}O_{2\max}$), 64 to 76% of maximum heart rate (HR_max_) or 12–13 on the rating of perceived exertion (RPE) scale [22]), or if exercise intensity was not reported, exercise described as running, cycling, or resistance training. Exclusion criteria were as follows: 1) studies conducted among populations with medical comorbidities or neuropsychiatric conditions that could impact angiogenesis markers (e.g. diabetes, schizophrenia, etc.) 2) mean age of study participants below 50 years of age; 3) significant study co-interventions that may impact the effect of exercise on angiogenesis protein concentrations (e.g. high altitude [23], blood flow restriction [24], or drug trials); or 4) exercise intensity below moderate intensity (e.g. yoga or easy walking).

When studies reported similar overlapping subjects, the larger sample size study was included. If an overlapping sample between studies was suspected, clarification was sought by emailing the corresponding authors. Studies with different populations or different exercise interventions in the same article were not pooled; a minimum of three studies are needed for a meta-analysis [25]. Studies where data cannot be extracted (e.g., pre- or post-intervention values not provided) were not included in a meta-analysis. If insufficient for a meta-analysis, results from the included studies were qualitatively summarized as numerically increased or decreased and whether the changes were significant. Inclusion eligibility was assessed by two independent reviewers, and conflicts were resolved by consensus between the reviewers.

### Data Extraction and Quality Assessment

Two independent reviewers extracted means and standard deviations (SD) of angiogenesis protein concentrations pre- and post-exercise, population characteristics (e.g. mean age, male proportion, body mass index (BMI), $\dot{V}O_{2\max}$), exercise intervention characteristics (e.g. exercise duration, intensity, and type/modality), risk of bias items (e.g. dropout rate, compliance), and other study details (e.g. compartment of blood concentrations, protein quantification assay) from included studies into a pre-formatted spreadsheet. When resting concentrations at multiple time points were available, the time-points that were closest to the initiation and completion of the exercise intervention were included for analysis. Mean and SD were estimated when the values were given in confidence intervals (CI), quartiles, standard errors, or graphic figures. Missing data were requested by email from the corresponding authors. Studies with different populations or different exercise interventions in the same article were analyzed separately. The risk of bias was evaluated based on criteria adapted from the Newcastle Ottawa Scale and the Cochrane Collaboration’s Risk of Bias Assessment Tool [26, 27]. Two independent reviewers assessed the risk of bias, and disagreement was resolved by consensus between the reviewers; the overall risk of bias was assessed based on whether the study methodology may greatly contribute to bias under the present review question.

### Statistical Analyses

Standardized mean differences (SMD; hedges’ g) with 95% CIs were calculated using the random-effects model as between-study variation was anticipated. Heterogeneity was quantified by the I^2^ statistic, and the Q statistics were used to test significant heterogeneity. If heterogeneity was detected (I^2^ > 50%), a pre-defined systematic investigation was performed using meta-regressions and subgroup analyses. Subgroup analyses were performed with 1) exercise type, 2) exercise duration, 3) population (healthy, overweight, and obese), 4) blood compartment, and 5) overall potential risk of bias rating. If more than 10 observations were available, meta-regressions with 1) mean age, 2) percent male, 3) exercise dose (calculated by multiplying the exercise duration per session, number of sessions per week, and number of weeks)[28], 4) BMI, and 5) $\dot{V}O_{2\max}$ were also performed. Publication bias was assessed using Egger’s test and funnel plots with trim-and-fill to adjust for the bias. Funnel plots were visually inspected for potential outliers which were analyzed using the leave-one-out meta-analysis method in STATA 17.0.

## Results

### Literature search findings

A total of 9288 records were found from the literature and two more were identified from references of included articles. Forty-four articles met the inclusion criteria with sufficient data [29-72], and 38 of them were included in the meta-analyses (Additional file 2). Seventeen angiogenesis markers were included in the qualitative synthesis (Table 1); five of which were included in meta-analyses: VEGF, endostatin, CD62E, FGF2, and MMP9. Characteristics of the included studies are presented in Additional file 3.

### Pre-and Post-intervention Comparisons

**Figure 1:**
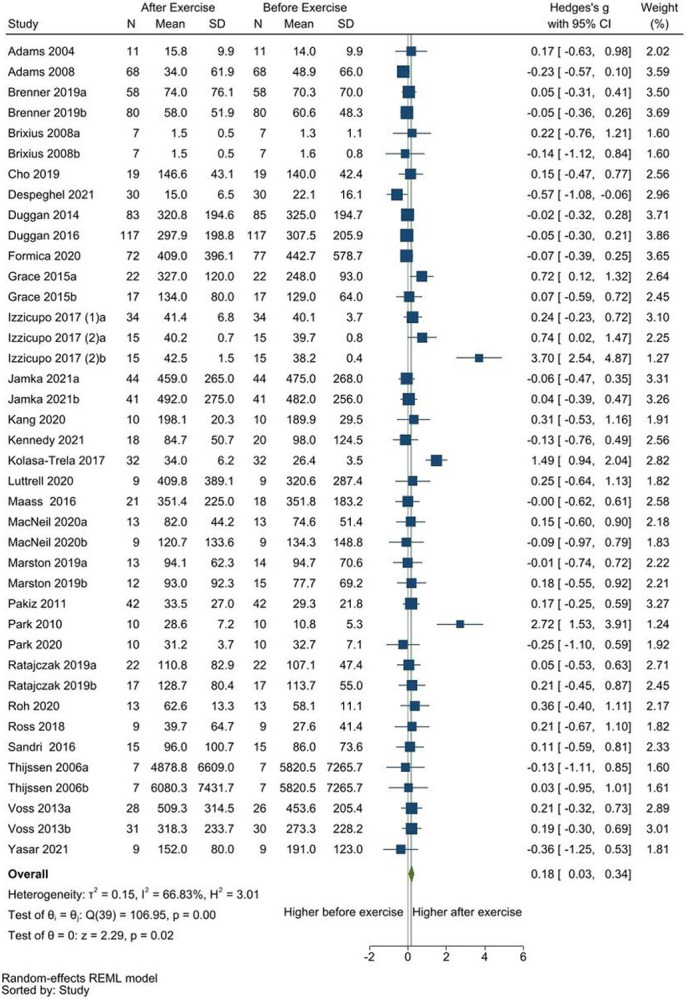
Meta-analysis of resting VEGF concentrations pre-and-post exercise. Higher VEGF was found after exercise.

**Figure 2:**
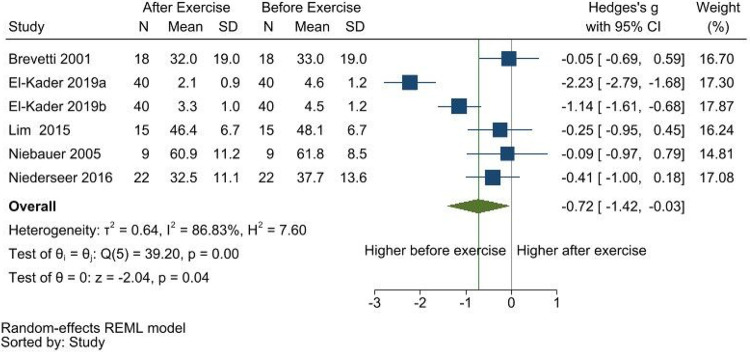
Meta-analysis of resting CD62E concentrations pre-and-post exercise. Higher CD62E was found before exercise.

In the meta-analyses (Table 2), resting blood concentrations of VEGF were significantly higher after exercise (SMD [95%CI] = 0.18 [0.03, 0.34], p = 0.02; Fig. 1), while CD62E concentrations were significantly lower after exercise (SMD [95%CI] = −0.72 [−1.42, −0.03], p = 0.04; Fig. 2). No differences in blood concentrations of endostatin (Additional file 4), FGF2 (Additional file 5), and MMP9 (Additional file 6), were found pre-and post-exercise. In the qualitative synthesis (Additional file 7), one article on ANGPTL4 and the one article on TSP-1 reported a significant increase after exercise. Other comparisons for ANGPTL2, ANGPTL4, HGF, PDGF-AA, PDGF-BB, VEGF-C, and VEGF-D were either not statistically significant or did not report statistical significance, but mostly showed an increasing direction after exercise. Although not statistically significant, MMP2 and PIGF comparisons showed a decreasing direction after exercise. The single study on angiogenin did not report a significant change with exercise.

### Assessment of potential bias

Thirty-five studies were found to have a low potential risk of bias and nine studies were found to have an unclear potential risk of bias mainly due to unclear descriptions of the 1) exercise interventions, 2) protein assays or measurement compartment, 3) mean age, 4) health status of the population (i.e. comorbidities), and/or 5) exercise adherence and dropout rate (Additional file 8). Egger’s test detected a significant risk of publication bias in VEGF (B[SE] = 1.91 [0.62], p = 0.002) and endostatin (B[SE] = −7.62 [1.90], p < 0.001), but publication bias was not detected (Egger’s test, p > 0.05) in CD62E, FGF2, and MMP9 (Additional files 9–13). Applying the trim-and-fill to the VEGF meta-analysis did not adjust the studies included or affect the effect size (SMD [95%CI] = 0.18 [0.03, 0.34]). Endostatin’s effect size remained non-significant after adjusting for potential publication bias using trim-and-fill (estimated SMD [95%CI] = 0.54 [−0.33, 1.41). Visual inspection of the VEGF funnel plot revealed three potential outliers (Additional file 9); the leave-one-out meta-analysis was performed and found similar effect sizes with the overall estimates for these three (“Kolasa-Trela 2017”: SMD[95%CI] = 0.09 [−0.02, 0.19], p = 0.1, “Izzicupo 2017 (2)b”: SMD [95%CI] = 0.12 [−0.003, 0.24], p = .06, and “Park 2010”: SMD[95%CI] = 0.14 [0.003, 0.28], p = 0.045; Additional file 14), as their updated estimates still fell within the 95% CI of the overall meta-analytical estimates (SMD [95%CI] = 0.18 [0.03, 0.34]).

### Exploration of heterogeneity

Significant heterogeneity was detected in meta-analyses (Table 2) for VEGF (I^2^ = 67% p < 0.001), CD62E (I^2^ = 87% p < 0.001), endostatin (I^2^ = 82% p < 0.001), and MMP9 (I^2^ = 89% p < 0.001), but not FGF2 (I^2^ = 0% p = 0.1). Subgroup analyses were performed for VEGF, CD62E, endostatin, and MMP9; meta-regressions were performed with VEGF.

#### VEGF

**Figure 3:**
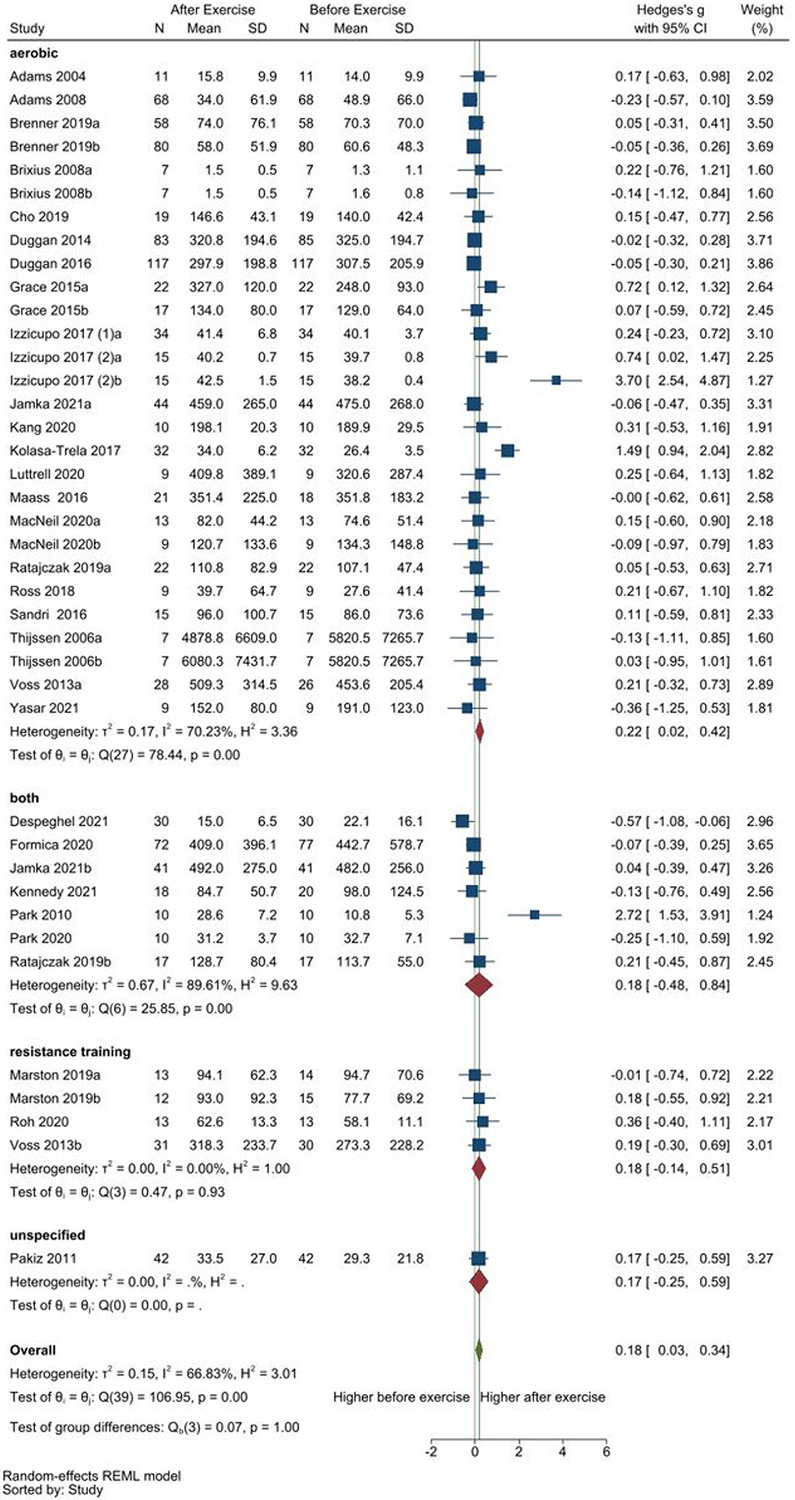
Sub-group analysis of exercise type on resting VEGF concentrations pre-and-post exercise. Studies either had aerobic exercise (SMD [95%CI] = 0.22 [0.02, 0.42], p = 0.03), resistance training (SMD [95%CI] = 0.18 [−0.14, 0.51], p = 0.3), a combination of both aerobic and resistance training (both: SMD [95%CI] = 0.18 [−0.48, 0.84], p = 0.6), or did not specify the exercise intervention (unspecified: only one comparison).

None of the subgroup analyses showed significant differences between the subgroups in VEGF (Fig. 3, Additional files 15–18). Subgroup analysis of exercise type showed higher VEGF after exercise only in aerobic exercise interventions (SMD [95%CI] = 0.22 [0.02, 0.42], p = 0.03) with no reduced heterogeneity (I^2^ = 70%, p < 0.001), but not in the resistance training or combined aerobic and resistance group (both; Fig. 3). Subgroup analysis of exercise duration (Additional file 15) did not show significant VEGF changes in the subgroup with at least 4 weeks of exercise (≥ 4 weeks) or single session of exercise (1 session); the ≥ 4 weeks subgroup showed a trend with higher VEGF after exercise (SMD [95%CI] = 0.13 [−0.003, 0.26], p = 0.06) and had reduced but still significant heterogeneity (≥ 4 weeks: I^2^ = 43% p < 0.001; 1 session: I^2^ = 69% p < 0.001). Subgroup analysis of the population (Additional file 16) also did not show significant VEGF changes in the healthy, overweight, and obese population subgroups, but the healthy subgroup showed a trend with higher VEGF after exercise (SMD [95%CI] = 0.21 [−0.004, 0.42], p = 0.05) with no reduced heterogeneity (healthy: I^2^ = 72%, p < 0.001; obese: I^2^ = 84% p < 0.001; overweight: I^2^ = 0%, p = 0.9). Similarly, subgroup analysis of the measurement compartment (Additional file 17) did not show significant VEGF changes in the plasma and serum subgroups. The plasma subgroup showed a trend (SMD [95%CI] = 0.42 [−0.02, 0.86], p = 0.06) but with no reduced heterogeneity (plasma: I^2^ = 89%, p < 0.001; serum: I^2^ = 0%, p = 0.96).

Subgroup analysis of the potential risk of bias (Additional file 18) showed higher VEGF after exercise only in the low potential risk of bias studies (SMD [95%CI] = 0.22 [0.04, 0.40], p = 0.01) with no reduced heterogeneity (I^2^ = 71%, p < 0.001), but not in the unclear potential risk of bias studies. Meta-regression analyses showed no significant associations between VEGF changes and mean age, percentage of the male population, exercise dose, baseline BMI, or baseline $\dot{V}O_{2\max}$ (Additional files 19–23).

#### CD62E

None of the subgroup analyses showed significant differences between the subgroups in CD62E (Additional files 24–27). No population subgroup analysis was performed since the population was healthy across all studies. CD62E was higher before exercise only in the ≥ 4 weeks exercise duration subgroup (SMD [95%CI] = −0.86 [−1.64, −0.08], p = 0.03) with no reduced heterogeneity (I^2^ = 88% p < 0.001) and in the serum compartment subgroup (SMD [95%CI] = −1.22 [−2.33, −0.11], p = 0.03) with no reduced heterogeneity (I^2^ = 91% p < 0.001). No significant changes in CD62E were found in the exercise type or the potential risk of bias subgroup that had more than one comparison.

#### Endostatin and MMP9

Endostatin was also analyzed with subgroups of exercise duration, population, measurement compartment, and potential risk of bias, yielding a similar division of the subgroups (Additional files 28–31) with significant between-group differences (χ^2^ = 18.4, p < 0.001); exercise type was aerobic across all studies, so no subgroup analysis was performed for exercise type. Among the 5 comparisons, endostatin was higher after exercise in the 1 session duration/serum compartment/low potential risk of bias subgroup (SMD [95%CI] = 0.97 [0.58, 1.36], p < 0.001) with reduced heterogeneity (I^2^ = 0% p = 0.7), but it seems to go in the opposite direction for the ≥ 4 weeks duration /plasma compartment/unclear potential risk of bias subgroup at a reduced heterogeneity (I^2^ = 0% p = 0.9) with the two comparisons included. MMP9 was also analyzed with subgroups of exercise type, duration, population, compartment, and potential risk of bias, showing no significant changes in the subgroups with more than one comparison (Additional files 32–36); none of the subgroup analyses showed significant differences between the subgroups that had more than one comparison.

## Discussion

This systematic review and meta-analysis aimed to examine the changes in angiogenesis markers with exercise in healthy older adults. Among the five angiogenesis markers that were included in meta-analyses, there were significant differences in VEGF and CD62E concentrations after exercise, but no differences in MMP9, FGF2, and endostatin. The increase in VEGF and the decrease in CD62E were heterogeneous with small and medium effect sizes, respectively. The high I^2^ values in VEGF and CD62E analyses suggest heterogeneity across studies, and the inconsistency in effect estimates may be contributed by differences between studies, such as demographic variables, exercise parameters, and measurement protocols. Many other proteins could not be included in the meta-analyses but showed a trend to increase with exercise in the qualitative analysis. Our findings supported the observation that exercise can induce angiogenesis, similar to previous reviews [11, 15, 16].

### VEGF

VEGF induces angiogenesis by activating VEGF receptors, particularly VEGF receptor-2, to increase microvascular permeability, endothelial cell proliferation and migration, and the release of matrix metalloproteinases [1]. A recent systematic review and meta-analysis on exercise and inflammatory markers evaluated VEGF, but only found one study from their search of randomized controlled trials, which is not enough for a meta-analysis [73]. Our meta-analysis found an increase in peripheral VEGF after exercise in exercise intervention studies. This result should be interpreted with caution as there might be potential outliers in the analysis, as suggested by the leave-one-out meta-analysis but not the standard trim- and-fill; the study characteristics of these comparisons did not reveal any noticeable differences in experimental protocols from other included studies. Although the connection between exercise-induced changes in peripheral VEGF and brain VEGF changes remains to be further investigated, animal studies showed that exercising skeletal muscle may induce VEGF and cerebral angiogenesis through the activation of lactate receptors [74, 75].

Our meta-regressions investigated the effects of demographic variables on the VEGF response. VEGF changes after exercise did not show a significant association with age or sex, although aging has been associated with decreased angiogenesis and VEGF levels [76], and females were found to have higher VEGF levels than males in adults and the elderly at baseline [77]. Although the meta-regression did not find BMI to be significantly associated with changes in VEGF concentrations after exercise, our subgroup analysis found changes in VEGF to have a trend only in the “healthy” and not overweight or obese population. Previously, serum VEGF levels were also positively correlated with BMI in non-diseased individuals [78]. Based on the current findings, age and sex may not affect the VEGF response after exercise in older adults, while it is unclear whether the response may be affected by BMI.

Variations in experimental protocols could also contribute to differences in VEGF response to exercise, as examined in our subgroup analyses. Our subgroup analyses also found that peripheral VEGF increased following aerobic exercise, but not after resistance training, similar to a previous meta-analysis which found increased peripheral BDNF after aerobic exercise but not resistance training [79]. Additionally, a previous review on exercise and angiogenesis suggested that although resistance and aerobic exercise both induce angiogenesis, the effects are stronger in aerobic exercise [11]; this could be attributed to the increased capillarity, particularly after aerobic training [80]. While there was no significant difference between the studies conducted in plasma or serum, changes in VEGF were found to have a trend only in studies conducted in plasma. Serum VEGF levels are significantly higher than plasma levels [81], and this difference could be attributed to the storage of VEGF within platelets [82] since serum VEGF levels could be increased by platelet activation in healthy and diseased states [83]. Although some evidence suggests that serum VEGF changes may be more useful than plasma VEGF in cancer patients for prognosis [83], the current evidence could not conclude whether plasma or serum is a more ideal specimen for monitoring VEGF changes with exercise in older adults. These findings suggest that the effects of exercise may differ due to cellular angiogenesis responses.

### CD62E

On the other hand, CD62E levels were found to decrease with exercise. CD62E is an adhesion molecule in vascular endothelial cells [84]. It is widely recognized as an endothelial and inflammatory marker regulating leukocyte accumulation, but it has been suggested to also mediate angiogenesis [85]. Although its role in angiogenesis remains to be clarified, the decreasing CD62E levels found with exercise in this paper may be explained by CD62E’s anti-angiogenic actions [5, 6]. Our current findings differ from a recent systematic review that did not find CD62E levels to change after low-to-moderate-intensity aerobic exercise and resistance exercise, although some other adhesion molecules in that review decreased [86]. This discrepancy may reflect the inclusion of newer studies in the current meta-analysis; also, the previous paper was a qualitative review [86] and not a meta-analysis. Our results are consistent with the notion that CD62E may be more responsive to long-term exercise, as our subgroup analysis showed significant changes only in the group that exercised for longer than 4 weeks. However, it should be noted that only one study was identified in the other exercise duration subgroup (i.e., 1 session group), thus more studies are needed to examine the effects of a single exercise session on peripheral CD62E levels. The CD62E response was also only significant in the serum subgroup and not the plasma subgroup, but this may be due to the similar inclusion of only 4-week exercise duration studies in the serum subgroup.

### Endostatin, FGF2, and MMP9

The other three proteins that were included in the meta-analyses, endostatin, FGF2, and MMP9, did not show significant changes in association with exercise, but they all had a small sample size. Notably, only three articles were found for endostatin; two of the articles had two different participant groups, and thus, it had five comparisons in total. Exploring further, the exercise duration, population (obesity), compartment, and quality assessment subgroup analyses all showed that one article was significantly different from the other two articles with significantly opposite endostatin responses. This finding suggests that exercise and population parameters may influence endostatin response to exercise, but more research would be needed. The current findings also suggest that FGF2, which can upregulate VEGF to induce angiogenesis [87], may not be activated by exercise; alternatively, besides FGF2, VEGF can be activated by other factors and other processes, such as hypoxia inducible factor[88]. Nevertheless, the findings should be interpreted with caution despite the low heterogeneity in its analysis, since similarly to the articles with endostatin, only three articles were found for FGF2. Moreover, MMP9 only had four comparisons in total from two articles. Three of the four articles also had an unclear risk of bias. Although the high heterogeneity was lowered in subgroup analyses, the subgroups often included only one or two comparisons. Considering the high heterogeneity and the limited number of articles in the current analyses, more research is needed to examine endostatin, FGF2, and MMP9 changes with exercise.

### Limitations and future directions

A few limitations should be considered when interpreting our findings. Many proteins could not be included in a meta-analysis due to the limited studies available. Amongst the five proteins that were included in the meta-analyses, only a few studies were identified for endostatin, FGF2, and MMP9. Thus, the small sample size may have contributed to the lack of response observed, and the results of endostatin, FGF2, and MMP9 should be interpreted with caution. In addition, despite attempts made to address the differences through subgroup and meta-regression analyses, variations across the studies in study parameters such as exercise interventions and demographics may have contributed to the high heterogeneity from the meta-analyses on VEGF, CD62E, endostatin, and MMP9. Furthermore, nine of forty-four studies included in this paper had an unclear overall risk of bias, and publication bias was a concern specifically in the VEGF meta-analysis where trim-and-fill was not able to adjust the bias. Also, the protein changes observed may not be specific to angiogenesis; changes in VEGF and CD62E could reflect changes in other pathways, like inflammation. For example, CD62E has also been shown to improve endothelial inflammation [86] and exercise is known to protect endothelial function by reducing inflammatory factors [89]. Therefore, more studies analyzing the effects of exercise on various angiogenesis markers are needed. Moreover, since some angiogenesis markers have shown transient responses after exercise [19], future research could be done on the duration of exercise’s effects on different angiogenesis proteins and the effects of measurement time points. Additionally, dehydration after exercise can also decrease blood volume, affecting blood concentrations [90], but the included studies in this meta-analysis did not provide enough information to examine this effect; future studies could further explore the effects of dehydration on angiogenesis levels.

## Conclusions

This systematic review and meta-analysis of angiogenesis blood markers in healthy older adults showed changes after exercise in some angiogenesis blood markers, including VEGF and CD62E. The increase in VEGF after exercise may differ depending on the exercise type, and potentially the blood compartment of measurements and population. The decrease in CD62E may also differ depending on exercise duration and blood compartment of measurements. Our findings suggest that multiple angiogenesis markers, including VEGF and CD62E, can change with physical exercise in older adults. However, many angiogenesis proteins were only qualitatively reviewed because they could not be included in meta-analyses, suggesting the need for further research. The study of additional angiogenesis blood markers can help elucidate the effects of exercise on angiogenesis.

## Figures and Tables

**Table 1:** Included proteins and their abbreviations (if applicable)

Protein Name	Abbreviation
Angiogenin	—
Angiopoietin-like protein 2	ANGPTL2
Angiopoietin-like protein 4	ANGPTL4
Endostatin	—
E-selectin	CD62E
Fibroblast growth factor 2	FGF2
Hepatocyte growth factor	HGF
Matrix metalloproteinase-2	MMP2
Matrix metalloproteinase-9	MMP9
Placental growth factor	PIGF
Platelet derived growth factor AA	PDGF-AA
Platelet derived growth factor BB	PDGF-BB
Thrombospondin-1	TSP-1
Placental growth factor	PIGF
Vascular endothelial growth factor	VEGF
Vascular endothelial growth factor C	VEGF-C
Vascular endothelial growth factor D	VEGF-D

**Table 2:** Summary of meta-analyses

Protein	Number of comparisons	SMD	[95% CI]	Z	p- value	I^2^ (%)	Chi- square	p- value	Potential Risk of publication bias
CD62E	6	−0.72	[−1.42, −0.03]	−2.04	0.04	86.8	39.2	< 0.001	Not detected
Endostatin	5	0.28	[−0.56, 1.11]	0.65	0.5	81.5	19.1	< 0.001	Detected
FGF2	5	0.03	[−0.18, 0.23]	0.24	0.8	0	7.3	0.1	Not detected
MMP9	6	−0.26	[−0.97, 0.45]	−0.71	0.5	89.1	30.2	< 0.001	Not detected
VEGF	40	0.18	[0.03, 0.34]	2.29	0.02	66.8	107.0	< 0.001	Detected

## Abbreviations

≥4 weeks: At least 4 weeks of exercise
1 session: Single session of exercise
ANGPTL2: Angiopoietin-like protein 2
ANGPTL4: Angiopoietin-like protein 4
BMI: Body mass index
CD62E: E-selectin
CI: Confidence interval
FGF2: Fibroblast growth factor 2
HGF: Hepatocyte growth factor
HR_max_: Maximum heart rate
HRR: Heart rate reserve
MMP2: Matrix metalloproteinase-2
MMP9: Matrix metalloproteinase-9
PDGF-AA: Platelet derived growth factor AA
PDGF-BB: Platelet derived growth factor BB
PIGF: Placental growth factor
RPE: Rating of perceived exertion
SD: Standard deviations
SMD: Standardized mean differences
TSP-1: Thrombospondin-1
VEGF: Vascular endothelial growth factor
VEGF-C: Vascular endothelial growth factor C
VEGF-D: Vascular endothelial growth factor D
$\dot{V}O_{2\max}$: Maximal oxygen consumption
$\dot{V}O_{2}R$: Oxygen consumption reserve
